# Toxicological Evaluation toward Refined Montmorillonite with Human Colon Associated Cells and Human Skin Associated Cells

**DOI:** 10.3390/jfb15030075

**Published:** 2024-03-20

**Authors:** Zhou Wang, Yibei Jiang, Guangjian Tian, Chuyu Zhu, Yi Zhang

**Affiliations:** Department of Inorganic Materials, School of Minerals Processing and Bioengineering, Central South University, Changsha 410083, China; zhouwang@csu.edu.cn (Z.W.); 215611016@csu.edu.cn (Y.J.); 215613081@csu.edu.cn (G.T.)

**Keywords:** refine, montmorillonite, toxicological evaluation, microscopic interaction, clinical medical orientation

## Abstract

Montmorillonite has been refined to overcome uncertainties originating from different sources, which offers opportunities for addressing various health issues, e.g., cosmetics, wound dressings, and antidiarrheal medicines. Herein, three commercial montmorillonite samples were obtained from different sources and labeled M1, M2, and M3 for Ca-montmorillonite, magnesium-enriched Ca-montmorillonite, and silicon-enriched Na-montmorillonite, respectively. Commercial montmorillonite was refined via ultrasonic scission-differential centrifugation and labeled S, M, or L according to the particle sizes (small, medium, or large, respectively). The size distribution decreased from 2000 nm to 250 nm with increasing centrifugation rates from 3000 rpm to 12,000 rpm. Toxicological evaluations with human colon-associated cells and human skin-associated cells indicated that side effects were correlated with excess dosages and silica sand. These side effects were more obvious with human colon-associated cells. The microscopic interactions between micro/nanosized montmorillonite and human colon-associated cells or human skin-associated cells indicated that those interactions were correlated with the size distributions. The interactions of the M1 series with the human cells were attributed to size effects because montmorillonite with a broad size distribution was stored in the M1 series. The M2 series interactions with human cells did not seem to be correlated with size effects because large montmorillonite particles were retained after refining. The M3 series interactions with human cells were attributed to size effects because small montmorillonite particles were retained after refining. This illustrates that toxicological evaluations with refined montmorillonite must be performed in accordance with clinical medical practices.

## 1. Introduction

Montmorillonite (MMT) is obtained from natural bentonite and used in various fields because of its natural abundance, cost-effectiveness, and nontoxic nature [1,2,3]. MMT has the chemical formula (Na, Ca)_0.33_(Al, Mg, Fe)_2_[(Si, Al)_4_O_10_](OH)_2_·4H_2_O, containing one Al-octahedral sheet and two Si-tetrahedral sheets. These elements, such as Si, Al, Fe, Ti, and Mn, could cause side effects in clinical medical studies, which might be due to silicon-containing minerals, iron-containing minerals, and titanium-containing minerals [4]. Several treatments and chemical methods have been used to refine MMT in laboratories and industry, such as sieving, sonication, sedimentation or centrifugation, and even long-term sedimentation combined with sonication and/or centrifugation [5,6]. In addition, selective dissolution under acidic or alkaline conditions was used to remove soluble salts, carbonates, organics, and toxic elements.

Refined MMT has been studied for various common health applications, such as cosmetics [7,8], wound dressings [9,10,11,12,13], and anti-diarrhea medicines [14], owing to the uniform bidimensional particle shapes, high length-to-height ratios, inherent stiffness, dual charge distribution, chemical inertness, biocompatibility, and active sites on the surface [15,16,17,18]. The results of these studies have attracted the interest of scientists and biotechnologists since micro/nanosized materials might also exhibit the same micro/nano biological interactions because one functional dimension is outside the nanoscale range of 1 nm to 100 nm, whereas organ-modified micro/nanosized materials might have uncertain effects on micro/nanobiological interactions [19,20,21,22]. The material parameters included size, shape, surface chemical characteristics, and surface topological structure, whereas the biological information included signaling molecule transmission, cell internalization, tissue distribution, and organ filtration [23,24,25,26]. Therefore, the refinement of MMT with a controllable length–radius and toxicological evaluation of refined MMT must be carried out in accordance with clinical methods, which will enable standardized evaluation in micro/nanobiological studies. Overall, the factors influencing MMT material information and cell biological information, such as the toxicological effects on human-derived cell lines and MMT–cell interactions, should be considered.

As shown in this manuscript, MMT refining for medical use required evaluation of trace impurities and size distribution, which were neglected in traditional MMT refining, i.e., the MMT content was the main criterion. Toxicological evaluations of refined MMT with human colon-associated cells and human skin-associated cells have been performed because oral administration and external use of MMT are the main medical utilization directions. Three commercial MMTs were described according to their sources, i.e., Ca-MMT (M1), magnesium-enriched Ca-MMT (M2), and silicon-enriched Na-MMT (M3). Commercial MMTs were refined through ultrasonic scission-differential centrifugation and divided into three categories. The material characteristics of the commercial MMT and the refined MMT were measured via X-ray fluorescence (XRF), X-ray diffraction (XRD), Fourier transform infrared (FTIR), differential scanning calorimetry (DSC-TG), scanning electron microscopy (SEM), and DSL. In addition, micro/nano MMT–human cell line interactions were investigated.

## 2. Materials and Methods

### 2.1. Materials

MMTs were obtained from Sand Technology Co., Ltd. (Ezhou, China) and labeled M1, M2, and M3 for Ca-MMT, magnesium-enriched Ca-MMT, and silicon-enriched Na-MMT. Human umbilical vein endothelial cells (HUVECs), human intestinal epithelial cells (HIEC6), and human colonic epithelial cells (NCM460) were obtained from the American Type Culture Collection (ATCC) (Manassas, VA, USA). Human immortalized keratinocytes (HaCaT) were obtained from Changsha Abiowell Biotechnology Co., Ltd. (Changsha, China). A Cell Counting Kit-8 (CCK-8) was obtained from Beijing Lablead Biotech Co., Ltd. (Beijing, China).

### 2.2. Processed MMT with Ultrasonic Scission-Differential Centrifugation

The MMT was refined through ultrasonic fission-differential centrifugation. In brief, 2.5 g of MMT was mixed with 50 mL of water to form an MMT suspension and sonicated at 750 W for 10 min. Subsequently, the sonicated MMT suspension was centrifuged at 3000, 6000, and 12,000 rpm to collect the sediment. The centrifuged supernatant was reserved for subsequent centrifugation. The Ca-MMT, magnesium-enriched Ca-MMT, and silicon-enriched Na-MMT were labeled M1, M2, and M3, respectively. Sonicated M1, M2, and M3 were centrifuged at 3000 rpm for 10 min to collect M(1,2,3)-L with large particles. Subsequently, the supernatant was recentrifuged at 6000 rpm for 10 min to collect the medium M(1,2,3)-M particles. The supernatant was recentrifuged at 12,000 rpm for 10 min to collect small M(1,2,3)-S particles. All sediments were cleaned with deionized water 1–3 times and then dried overnight at 60 °C.

### 2.3. Characterization

Scanning electron microscopy (SEM) images were recorded using an MIRA3 LMU scanning electron microscope from Tescan (Brno, Czech Republic). The vacuum pressure was 5 × 10^−3^ Pa, and the accelerating voltage was 20 kV. All samples were suspended in deionized water at a concentration of 1 mg/mL, and then the solutions were dripped onto silicon slices and freeze-dried for 2 h. The X-ray diffraction (XRD) data were recorded using a TD-3500 instrument from Dandong Tongda Science & Technology Co., Ltd. (Dandong, China). The wavelength of the copper anode was λ(Kα) = 0.15406 nm. Fourier transform infrared (FTIR) spectra were recorded using a TENSOR II instrument from Bruker (Berlin, Germany). The spectra were scanned over the range of 400 to 4000 cm^−1^. X-ray fluorescence (XRF) data were recorded using an Axios mAX wavelength dispersive XRF spectrometer from PANalytical B.V. (Almelo, The Netherlands).

### 2.4. Toxicological Evaluation and Interaction Observation

Cell culture. Human skin-associated cell lines were incubated at 37 °C with 5% CO_2_ in DMEM containing 10% FBS and 1% Pen/Strep, i.e., the HUVEC line and HaCaT cell line. Human colon-associated cells were incubated at 37 °C with 5% CO_2_ in RPMI 1640 medium supplemented with 10% FBS and 1% Pen/Strep, i.e., the HIEC6 cell line and the NCM460 cell line. Single cells were isolated from 0.25% trypsin-EDTA solutions and cultured in the medium.

Toxicological evaluation. The cell lines were incubated for 12–24 h in 96-well cell culture plates (2 × 10^3^ cells/well) with different culture media and then incubated for 24 h in fresh culture media supplemented with MMT solutions at concentrations ranging from 0 to 300 μg/mL. CCK-8 was used to evaluate the cell survival rate by detecting formazan (450 nm) produced by the reduction of dehydrogenase in living cells. Cell viability was calculated as follows:

Cell viability = (OD_experiment_)/(OD_Control_) × 100%

Interaction observations. The cell lines were incubated for 12–24 h in 12-well cell culture plates (1 × 10^4^ cells/well) with different culture media and then incubated for 24 h in a fresh culture medium containing 200 μg/mL MMT solution. The treated cell lines were fixed for 24 h in 2.5% glutaraldehyde and then dehydrated in a graded ethanol series for SEM observation.

### 2.5. Statistical Analyses

The mean and standard deviation (S.D.) were calculated from the original data. All studies were conducted with three independent biological replicates. Statistical analyses were conducted with ANOVA and subsequent Student’s *t* tests. *p*-values less than 0.05, 0.005, and 0.0005 were considered significant at the *, **, and *** levels, respectively.

## 3. Results and Discussion

### 3.1. Material Characterization and Toxicological Evaluation of MMT

The XRF results for the MMTs from different sources are shown in Table 1. Ca-MMT and Na-MMT had different Ca and Na contents. The chemical contents in M1 decreased in the order Si, Al, Mg, Ca, Fe, F, Na, K, Ti, etc. The high Si content in M1 was attributed to silicon-containing minerals. The chemical element contents in M2 decreased in the order Si, Mg, Al, Fe, Ca, Na, F, K, Cl, Ti, etc. The high Mg content and Si content in M2 were attributed to magnesium-containing minerals and silicon-containing minerals. The element contents in M3 decreased in the order Si, Al, Mg, Fe, Na, Ca, Ti, F, K, S, etc. The high Si content and Fe content in M3 were attributed to silicon-containing minerals and iron-containing minerals. Metal ions such as Ca, Fe, and Zn are essential trace elements associated with human health. Ca intake, according to the WHO recommendation, should be 1000–1200 mg/d; the blood Ca concentration in adults is approximately 2.25–2.75 mmol/L; the total Ca content in adults is 850–1200 kg; and excess calcium intake or overload of Ca stores could induce diseases due to broken ion homeostasis. According to the WHO recommendations, the Fe intake should be 8–10 mg/d, the blood Fe concentration in adults is approximately 1.1–1.6 mol/L, the total Fe content in adults is 4–5 g, and excess Fe intake or overload of iron stores could induce diseases due to reactive •OH radicals. The Zn intake, according to the WHO recommendation, should be 8–11 mg/d; the blood Zn concentration in adults is approximately 11–18 μmol/L; the total Zn content in adults is 2–3 g; and excess Zn intake or overload of Zn stores could induce diseases due to disrupted ion homeostasis and impaired immune function. In addition, metal ions with different hemostatic mechanisms are used in wound healing; for example, Ca accelerates thrombosis by facilitating the conversion of prothrombin to thrombin, Fe stabilizes thrombin and plays a role in accelerating blood coagulation, and Zn is involved in all stages of wound healing.

**Figure 1:**
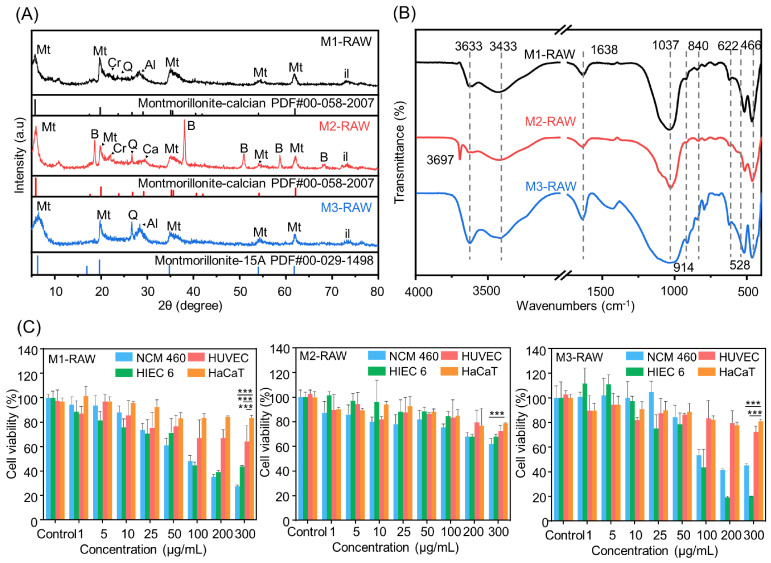
Characterization and toxicological evaluation of MMT via (**A**) XRD, (**B**) FTIR, and (**C**) CCK-8. M1-RAW: unrefined Ca-MMT; M2-RAW: unrefined magnesium-enriched Ca-MMT; M3-RAW: unrefined silicon-enriched Na-MMT. The P value was determined by ANOVA and Student’s *t* tests. *** *p* < 0.0005.

The XRD patterns of the MMT samples from different sources are shown in Figure 1A. According to the M1 XRD results in Supplementary Materials Table S1, reflections were observed at 19.79°, 35.17°, 53.98°, and 61.88°, which were consistent with the Ca-MMT standard card (JCPDS Card No. 00-058-2007). The strongest reflection was located in the 4–8° range, which was ascribed to the 001 plane in MMT, and the basal spacing, *d*_001_, was calculated as approximately 1.48 nm. These reflections were observed at 21.86°, 22.75°, 27.97°, and 73.02°, which were consistent with the standard card for cristobalite (JCPDS Card No. 00-039-1425), the standard card for quartz (JCPDS Card No. 01-070-2537), the standard card for albite (JCPDS Card No. 00-010-0393), and the standard card for illite (JCPDS Card No. 00-029-1496). According to the M2 XRD results in Table S2, reflections were observed at 19.79°, 35.08°, 53.95°, and 62.09°, which were consistent with the Ca-MMT standard card (JCPDS Card No. 00-058-2007). The strongest reflection was located in the 4–8° range, which was ascribed to the 001 plane in MMT, and the basal spacing, *d*_001_, was approximately 1.49 nm. These reflections were observed at 18.61°, 38.00°, 50.82°, 58.66°, and 68.35°, which were consistent with the standard card for brucite (JCPDS Card No. 00-007-0239). These reflections were observed at 21.97°, 26.68°, 29.51°, and 73.02°, which were consistent with the standard card for cristobalite (JCPDS Card No. 00-039-1425), the standard card for quartz (JCPDS Card No. 00-033-1161), the standard card for calcite (JCPDS Card No. 00-047-1743), and the standard card for illite (JCPDS Card No. 00-029-1496). According to the M3 XRD results in Table S3, reflections were observed at 19.86°, 34.85°, 54.08°, and 62.01°, which were consistent with the Na-MMT standard card (JCPDS Card No. 00-029-1498). The strongest reflection was located in the 4–8° range, which was ascribed to the 001 plane in MMT, and the basal spacing, *d*_001_, was approximately 1.37 nm. These reflections were observed at 26.74°, 28.07°, and 73.04°, which were consistent with the quartz standard card (JCPDS Card No. 01-070-2537), albite standard card (JCPDS Card No. 00-010-0393), and illite standard card (JCPDS Card No. 00-029-1496), respectively. Therefore, these XRD patterns indicated that MMT was the dominant mineral. The impurities include brucite, quartz, cristobalite, calcite, albite, illite, etc., which can cause side effects in humans. For example, quartz enhances the production of reactive oxygen species and results in oxidative damage [27,28,29].

The FTIR spectra of MMT from different sources are shown in Figure 1B. The structural O–H stretching vibration of alumina was located at approximately 3633 cm^−1^, the O–H stretching vibration of water was located at approximately 3433 cm^−1^, the O–H deformation vibration of water was located at approximately 1638 cm^−1^, and the O–H deformation vibration at approximately 1638 cm^−1^ was correlated with the water content in MMT, indicating a high water content in M3. For the Si–O bonds in MMT, the Si–O–Si stretching vibration was located at approximately 1037 cm^−1^, the Si–O–Si stretching vibration was located at approximately 622 cm^−1^, the Si–O–Al deformation vibration was located at approximately 523 cm^−1^, and the Si–O–Si deformation vibration was located at approximately 466 cm^−1^. For the Al–O bonds in MMT, the Al–Al–OH deformation vibration band was located at approximately 914 cm^−1^, the Al–Fe–OH deformation vibration band was located at approximately 840 cm^−1^, and the strength of the Al–Fe–OH deformation vibration band was correlated with the Fe content in MMT, indicating a high Fe content in M3. In addition, the Si–O stretching vibration was located at approximately 840 cm^−1^ and attributed to silica sand, and the Mg–OH stretching vibration was located at approximately 3697 cm^−1^ and attributed to brucite, which was observed in M2. Therefore, the FTIR data indicated that MMT contained Si–OH and Al–OH, and Si–OH or Al–OH at the structural or basal sites would cause constant charges, while surface -OH at edge sites would cause variable charges. Additionally, the net negative charge could facilitate MMT interactions with cationic molecules, and contact with opposite charges could facilitate MMT–cell interactions.

Toxicological evaluations of MMT from different sources with human colon-associated cells and human skin-associated cells are shown in Figure 1C. For M1, the cell survival rate of the HaCaT cell line was above 80% until the maximum amount was added; the cell survival rate of the HUVEC line was close to 80% with 50 μg/mL M1 treatment and decreased at a constant rate until the maximum addition, whereas the survival rates of the NCM460 cell line and HIEC-6 cell line were close to 80% after 25 μg/mL M1 treatment and decreased rapidly until the maximum addition. For M2, the survival rates of HaCaT cells and HUVECs were greater than 80% until maximum addition, and the survival rates of NCM460 cells and HIEC-6 cells were greater than 80% before treatment with 200 μg/mL M2. For M3, the survival rates of HaCaT cells and HUVECs were greater than 80% until the maximum addition, whereas the survival rates of the NCM460 cells and HIEC-6 cells were approximately 80% after treatment with 50 μg/mL M3, followed by a sudden decrease after treatment with 100 μg/mL M3. These results indicated that the side effects seem to correlate with excess dosages and the amounts of silica sand, and they were much more obvious in human colon-associated cells than in human skin-associated cells. In addition, MMT and brucite were harmless to human colon-associated cells and human skin-associated cells. Therefore, MMT refining must be carried out in accordance with clinical practices, which would overcome the uncertainties originating from different sources.

### 3.2. Material Characterization of Refined MMT

**Figure 2:**
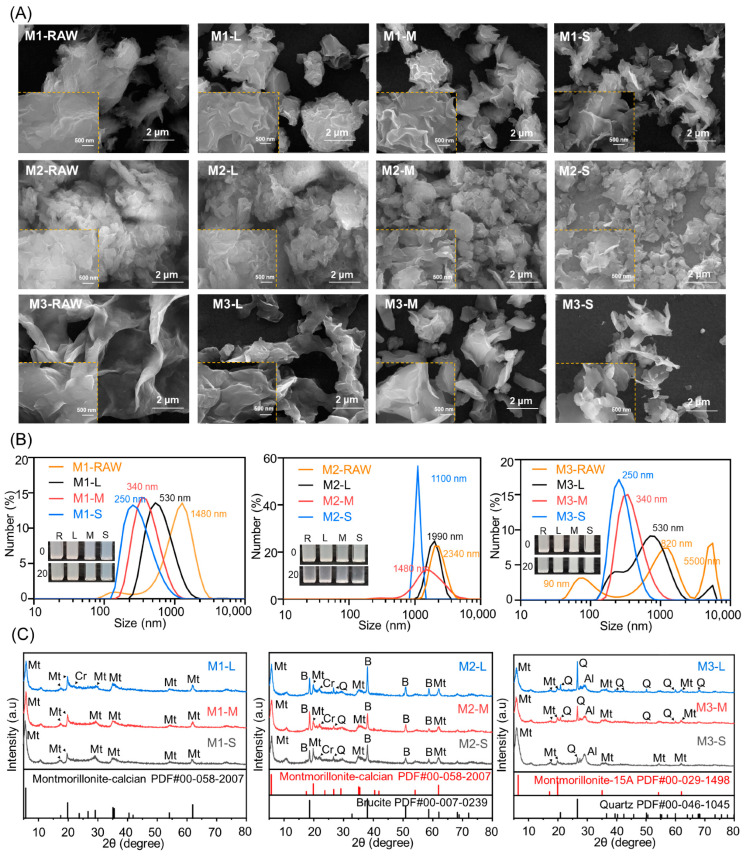
Characterization of refined MMT via (**A**) SEM, (**B**) DSL, and (**C**) XRD. The refined MMTs with small, medium, and large particle sizes were labeled S, M, and L, respectively.

SEM images of MMTs from different sources are shown in Figure 2A, Figures S1 and S2. The MMT aggregates with multilateral rhombus shapes formed inhomogeneous MMT sheets. Refined MMTs with homogeneous shapes and controllable sizes were obtained through ultrasonic fission-differential centrifugation, and the large MMT sheets were still aggregated and had thick laminated structures. Medium-sized MMT sheets were scattered and had a thin laminated structure. Small MMT sheets were exfoliated and had an ultrathin laminated structure. In addition, the average thickness of the MMT sheets decreased with an increasing centrifugation rate from 3000 rpm to 12,000 rpm.

The size distributions of the refined MMT prepared with centrifugation rates ranging from 3000 rpm to 12,000 rpm are shown in Figure 2B. For the M1 series, the centric size distribution ranged from 0.1 to 5.0 μm, and the average size was close to 1300 nm. The average size of refined M1 decreased from 500 nm to 250 nm with an increasing centrifugation rate. In addition, the centric size distributions of the widths and heights seemed almost the same in the M1 series. For the M2 series, the centric size distribution ranged from 1.0 to 5.0 μm, and the average size was close to 2000 nm. The average size of refined M2 decreased from 2300 nm to 1100 nm with an increasing centrifugation rate. In addition, the centric size distribution narrowed with decreasing average size. For the M3 series, the tricentric size distribution for M3 ranged from 0.2 nm to 9.0 μm, the tricentric size distribution for M3-L ranged from 0.1 nm to 7.0 μm, the centric size distribution for refined M3-M ranged from 0.1 nm to 5.0 μm with an average size close to 350 nm, and the centric size distribution for refined M3-S ranged from 0.1 nm to 1.0 μm with an average size close to 250 nm. In addition, the centric size distributions narrowed with decreasing average sizes. These results indicated that the size distributions decreased with increasing centrifugation rates from 3000 rpm to 12,000 rpm, and MMT was refined to give different size distributions through ultrasonic fission-differential centrifugation.

The XRD patterns of the refined MMT are shown in Figure 2C. For the M1 series, reflections for Ca-MMT, cristobalite, and albite were observed in the XRD patterns. The (003) reflection of Ca-MMT was visible in the M1 series, which was attributed to its content and instrumental characteristics. The (101) reflection of cristobalite was almost indiscernible with a decreasing size distribution, which was attributed to its content. The (002) reflection for albite broadened with decreasing size distribution, which was attributed to structural defects. The (117) reflection of illite was almost indiscernible in the M1 series, which was attributed to the content and instrumental characteristics. For the M2 series, reflections for Ca-MMT, brucite, cristobalite, quartz, and calcite were observed in the XRD patterns. The (100) peak for the Ca-MMT increased with decreasing size distribution, which was attributed to the presence of an intact structure. The (101) reflection of cristobalite broadened with decreasing size distribution, which was attributed to structural defects. The intensities of these brucite reflections increased with decreasing size distribution, which was attributed to the brucite content and particle sizes. The (117) reflection of illite was almost indiscernible in the M2 series, which was attributed to its content and instrumental characteristics. For the M3 series, the reflections of Na-MMT, quartz, and albite were observed in the XRD patterns. The (001) peak for Na-MMT increased with decreasing size distribution, which was attributed to an intact structure. The intensities of the reflections for the quartz grains increased with decreasing size distribution, which was attributed to the quartz content and particle sizes. The (002) reflection for albite increased with decreasing size distribution, which was attributed to the intact structure. The (117) reflection of illite was almost indiscernible in the M1 series, which was attributed to the content and instrumental characteristics. Therefore, the XRD results indicated that some impurities were difficult to remove from the refined MMT, and these impurities did not seem to correlate with the size distribution. In addition, selective dissolution under acidic or alkaline conditions was used to remove soluble salts, carbonates, organics, and toxic elements.

### 3.3. Toxicological Evaluation of the Refined MMT

**Figure 3:**
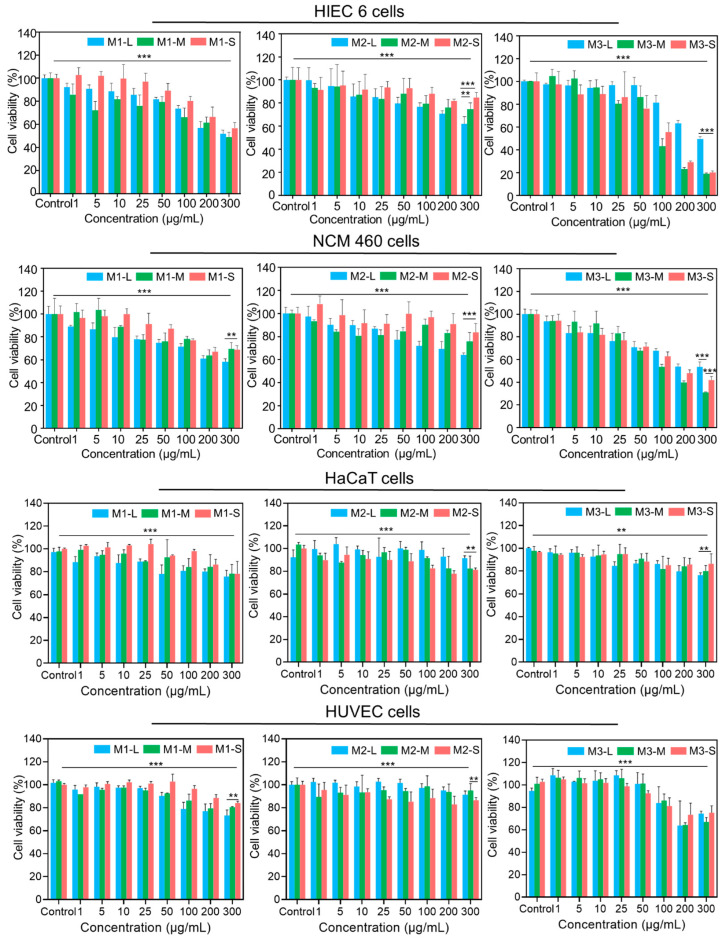
Toxicological effects of refined MMT on human colon-associated cells (HIEC 6 and NCM460) and human skin-associated cells (HaCaT and HUVECs). The *p* value was determined by ANOVA and Student’s *t* tests. ** *p* < 0.005, *** *p* < 0.0005.

Toxicological evaluations of refined MMT with human colon-associated cells and human skin-associated cells are shown in Figure 3. For human colon-associated cells, the cell survival rate with the refined M1 series was close to 80% with 50 μg/mL refined M1 and then decreased at a flat rate until maximum addition. The cell survival rate with the refined M2 series was close to 80% with the 100 μg/mL treatment, and the cell survival rate for the refined M2-S was above 80% until maximum addition. The cell survival rate for the refined M3 series was close to 80% with the 50 μg/mL treatment and then decreased rapidly until the maximum addition. For human skin-associated cells, the cell survival rate with the refined M1 series was greater than 80% until maximum addition, the cell survival rate with the refined M2 series was greater than 80% until maximum addition, and the cell survival rate with the refined M3 series was greater than 80% until maximum addition, but we excluded the HUVEC line with the 200 μg/mL M3 series treatment. These results indicated that the impurities were correlated with excess dosages and the amount of silica sand, and they were much more obvious in the human colon-associated cells than the human skin-associated cells. In addition, MMT and brucite are harmless to human colon-associated cells and human skin-associated cells.

### 3.4. MMT–Human Cell Interactions

**Figure 4:**
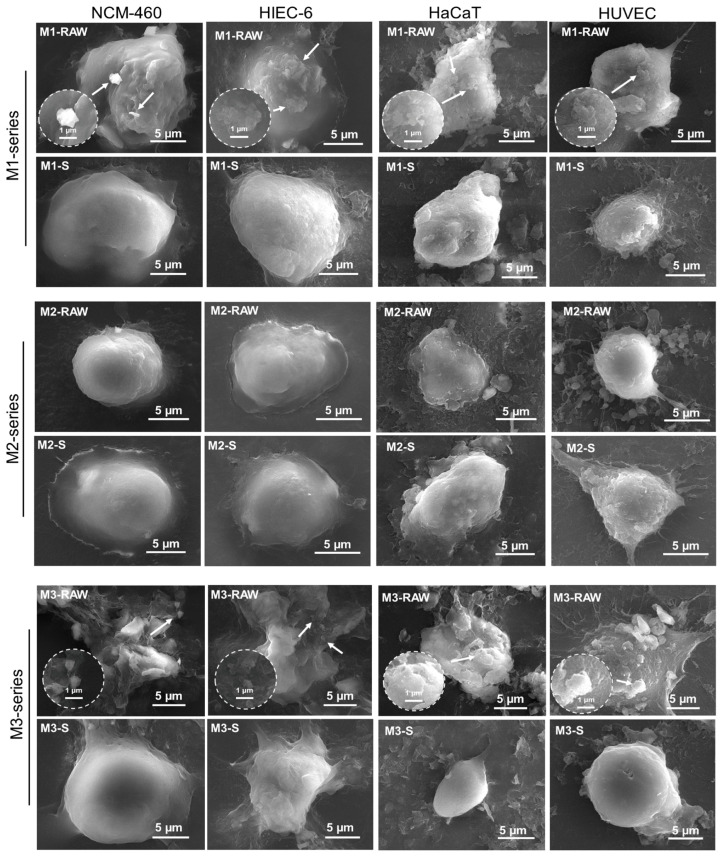
SEM images of MMT–human cell interactions. Unrefined Ca-MMT was labeled M1-RAW, and refined M1-RAW with small particle sizes was labeled M1-S. Unrefined magnesium-enriched Ca-MMT was labeled M2-RAW, and refined M2-RAW with small particle sizes was labeled M2-S. Unrefined silicon-enriched Na-MMT was labeled M3-RAW, and refined M3-RAW with small particle sizes was labeled M3-S. The thumbnail showed an enlarged image featuring a white arrow indicating the presence of MMT.

SEM images of MMT–human cell interactions are shown in Figure 4. In the literature, the cellular morphologies in SEM images were described as round and slender. The cell surface exhibited a negative electrostatic charge attributed to the presence of glycoproteins on the membrane. Upon stimulation, the cell surface underwent a transition from smooth to rough and experienced a reduction in mucus secretion [30,31]. The microscopic interactions between micro/nanosized MMT and human colon-associated cells or human skin-associated cells indicated that these side effects were correlated with the size distribution. Notably, MMT-RAW was internalized with an undispersed agglomerated morphology, which caused an increase in the MMT particle sizes. This interaction may have caused the aggregation of MMT-RAW into larger particles, resulting in increased cytotoxicity. The human cells interacted with M1 more than with M1-S, which was attributed to the broader size distribution and smaller sizes. In addition, mucus secretion resulting from mechanical stimuli was observed in the M1 series–human skin-associated cell interactions. For M2 and M2-S, human colon-associated cells seemed to have no response to the M2 series, which was attributed to large particle sizes, whereas human skin-associated cells interacted with the M2 series due to mechanical stimulus-induced mucus secretion. In addition, microscopic interactions with M2 featuring a small size distribution were more obvious and more uniform with human skin-associated cells. For M3 and M3-S, the human cells interacted with M3 more than with M3-S, which was attributed to more small MMT particles stored in M3. Therefore, the M1 series–human cell interactions were attributed to size effects because MMTs with broader size distributions were stored in the M1 series, and the M2 series–human cell interactions did not seem to correlate with size effects because large MMTs were stored in the M2 series. The M3 series–human cell interactions were attributed to size effects because small MMTs were stored in the M3 series.

## 4. Conclusions

Refinement of MMT has been used to overcome uncertainties originating from different sources and offers opportunities for addressing various common health issues. Refined MMT with different particle size distributions was obtained through ultrasonic scission-differential centrifugation, and some harmful chemical elements were difficult to remove from the refined MMT. The characterization data indicated that the size distribution decreased with increasing centrifugation rate, while the structural morphology changed from aggregates with a thick laminated structure to an exfoliated ultrathin laminated structure. Toxicological evaluations with human colon-associated cells and human skin-associated cells indicated that the cell survival rates with MMT were not correlated with the size distribution, whereas side effects were correlated with excess dosages and silica sand. Instead, the microscopic interactions between micro/nanosized MMT and human colon-associated cells or human skin-associated cells indicated that those interactions were correlated with the size distributions. Therefore, refinement of MMT must be carried out in accordance with clinical medical practices, i.e., those for cosmetics, wound dressings, and anti-diarrhea medicines, to overcome the uncertainties originating from different sources.

## Figures and Tables

**Table 1:** Characterization evaluation of MMT via XRF.

	Oxide Contents by Analysis, wt %												
Sample	SiO_2_	Al_2_O_3_	MgO	Na_2_O	Fe_2_O_3_	CaO	TiO_2_	MnO	K_2_O	ZrO_2_	SO_3_	Cl	F
M1-RAW	65.554	20.309	7.400	0.568	2.001	2.575	0.156	0.071	0.286	0.014	0.034	0.027	0.955
M2-RAW	55.685	14.886	23.884	0.630	1.787	1.609	0.134	0.031	0.290	0.012	0.092	0.302	0.613
M3-RAW	61.369	20.793	4.860	3.757	3.940	2.493	1.410	0.010	0.228	0.025	0.371	0.059	0.535

## Data Availability

The original contributions presented in the study are included in the article/supplementary material, further inquiries can be directed to the corresponding authors.

## Supplementary Materials

The following supporting information can be downloaded at https://www.mdpi.com/article/10.3390/jfb15030075/s1, Figure S1: SEM images of refined MMT at 50 k× and 100 k× magnification. Figure S2: SEM images of refined MMT at magnifications of 2 k× and 20 k×. Table S1: XRD data for M1. Table S2: XRD data for M2. Table S3: XRD data for M3.
