# Supplementary material for: Toxicological Evaluation toward Refined Montmorillonite with Human Colon Associated Cells and Human Skin Associated Cells
Source: J Funct Biomater. 2024 Mar 20;15(3):75. doi: 10.3390/jfb15030075 (PMC10971592; doi:10.3390/jfb15030075)
Supplement: Supplementary file 1 [file jfb-15-00075-s001.zip › jfb-2919696-supplementary.pdf]

Supporting Information

Toxicological evaluation toward refined montmorillonite with human colon associated cells and human skin associated cells

Zhou Wang[1#], Yibei Jiang[1#], Guangjian Tian[1], Chuyu Zhu[1*], Yi Zhang[1*]

1 Department of Inorganic Materials, School of Minerals Processing and Bioengineering, Central South University, Changsha 410083, China

# These authors contributed equally: Zhou Wang, Yibei Jiang

* Corresponding authors: Chuyu Zhu (email: zhucyu@csu.edu.cn); Yi Zhang (email: yee_z10@csu.edu.cn).

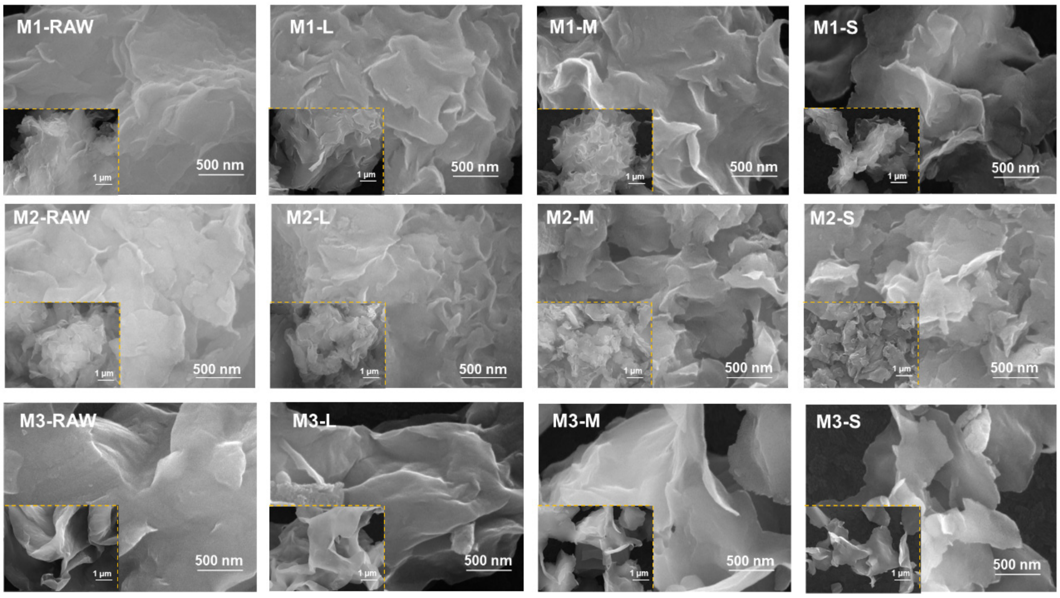

Figure S1 SEM toward refined montmorillonite with 50k× and 100k× magnification.

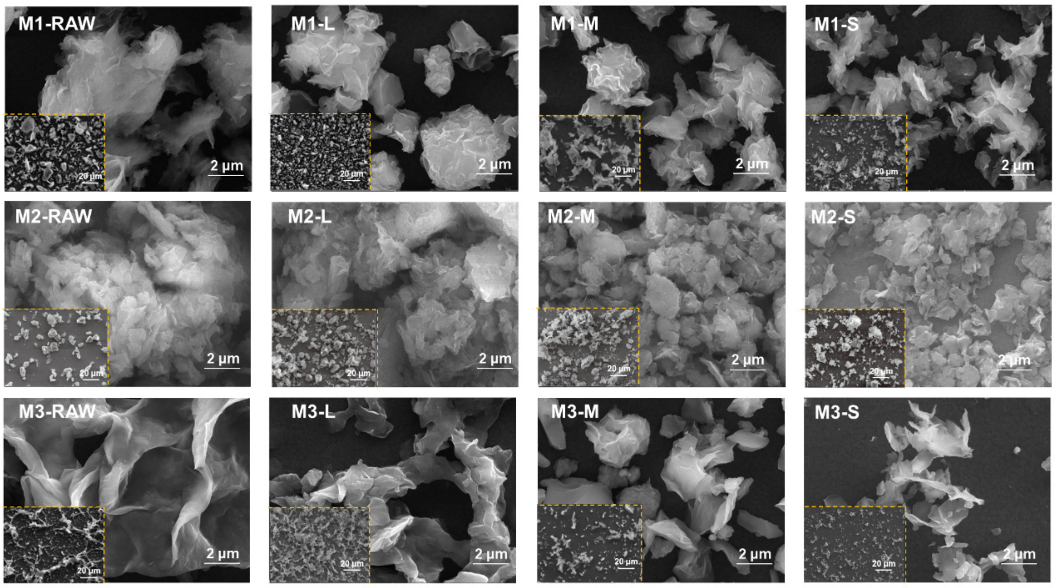

Figure S2 SEM toward refined montmorillonite with 2k× and 20k× magnification.

Table S1 XRD data toward M1

Mineral	Chemical Formula	JCPDS Card No.	2θ	h k l
Ca-montmorillonite	$Ca_{0.2}(Al,Mg)_2Si_4O_{10}(OH)_2$	00-058-2007	5.84	001
Ca-montmorillonite	$Ca_{0.2}(Al,Mg)_2Si_4O_{10}(OH)_2$	00-058-2007	19.79	100
Ca-montmorillonite	$Ca_{0.2}(Al,Mg)_2Si_4O_{10}(OH)_2$	00-058-2007	35.17	110
Ca-montmorillonite	$Ca_{0.2}(Al,Mg)_2Si_4O_{10}(OH)_2$	00-058-2007	53.98	210
Ca-montmorillonite	$Ca_{0.2}(Al,Mg)_2Si_4O_{10}(OH)_2$	00-058-2007	61.88	300
Cristobalite	SiO_2	00-039-1425	21.86	101
Quartz low	SiO_2	01-070-2537	22.75	101
Albite	$Na(Si_3Al)O_8$	00-010-0393	27.97	011
Illite-1M	$K_{0.7}Al_2(Si,Al)_4O_{10}(OH)_2$	00-029-1496	73.02	117

Table S2 XRD data toward M2

Mineral	Chemical Formula	JCPDS Card No.	2θ	h k l
Ca-montmorillonite	$Ca_{0.2}(Al,Mg)_2Si_4O_{10}(OH)_2$	00-058-2007	5.89	001
Ca-montmorillonite	$Ca_{0.2}(Al,Mg)_2Si_4O_{10}(OH)_2$	00-058-2007	19.79	100
Ca-montmorillonite	$Ca_{0.2}(Al,Mg)_2Si_4O_{10}(OH)_2$	00-058-2007	35.08	110
Ca-montmorillonite	$Ca_{0.2}(Al,Mg)_2Si_4O_{10}(OH)_2$	00-058-2007	53.95	210
Ca-montmorillonite	$Ca_{0.2}(Al,Mg)_2Si_4O_{10}(OH)_2$	00-058-2007	62.09	300
Brucite	$Mg(OH)_2$	00-007-02339	18.61	001
Brucite	$Mg(OH)_2$	00-007-02339	38.00	101
Brucite	$Mg(OH)_2$	00-007-02339	50.82	102
Brucite	$Mg(OH)_2$	00-007-02339	58.66	110
Brucite	$Mg(OH)_2$	00-007-02339	68.35	103
Cristobalite	SiO_2	00-039-1425	21.97	101
Quartz	SiO_2	00-033-1161	26.68	101
Calcite	$CaCO_3$	00-047-1743	29.51	104
Illite-1M	$K_{0.7}Al_2(Si,Al)_4O_{10}(OH)_2$	00-029-1496	73.02	117

Table S3 XRD data toward M3

Mineral	Chemical Formula	JCPDS Card No.	2θ	h k l
Na-montmorillonite	$Na_{0.3}(Al,Mg)_2Si_4O_{10}(OH)_2{\bullet}4H_2O$	00-029-1498	6.45	001
Na-montmorillonite	$Na_{0.3}(Al,Mg)_2Si_4O_{10}(OH)_2{\bullet}4H_2O$	00-029-1498	19.86	100
Na-montmorillonite	$Na_{0.3}(Al,Mg)_2Si_4O_{10}(OH)_2{\bullet}4H_2O$	00-029-1498	34.85	105
Na-montmorillonite	$Na_{0.3}(Al,Mg)_2Si_4O_{10}(OH)_2{\bullet}4H_2O$	00-029-1498	54.08	210
Na-montmorillonite	$Na_{0.3}(Al,Mg)_2Si_4O_{10}(OH)_2{\bullet}4H_2O$	00-029-1498	62.01	300
Quartz	SiO_2	00-046-1045	26.74	101
Albite	$Na(Si_3Al)O_8$	00-010-0393	28.07	002
Illite-1M	$K_{0.7}Al_2(Si,Al)_4O_{10}(OH)_2$	00-029-1496	73.04	117
